# Learning to serve: social learning and organizational customer orientation–employee job performance link in Nigerian service firms

**DOI:** 10.3389/fpsyg.2025.1666546

**Published:** 2025-12-08

**Authors:** Da Teng, Wei Song, Weiyue Wang

**Affiliations:** 1 Beijing University of Chemical Technology, Beijing, China; 2 Coventry University, Coventry, United Kingdom; 3 University of Birmingham Business School, Birmingham, United Kingdom

**Keywords:** organizational customer orientation, employee customer orientation, openness to experience, job performance, social learning theory

## Abstract

Organizational Customer Orientation (OCO) refers to an organization’s continuous and proactive disposition that puts the interests of customers first. Extant studies have explored the relationship between OCO and employee job performance. However, the results are mixed and inconsistent. Collecting a set of multisource data at different points of times in Nigeria, this research advances the literature by arguing that Employee Customer Orientation (ECO) acts as a mechanism between OCO and employee job performance. Moreover, we argue that this mediation effect is moderated by the employee’s personality trait of openness to experience. The results support the view that, as expected, ECO mediates the effect of OCO on employee job performance. And, this mediating effect is moderated by the employee’s openness to experience. From a managerial perspective, our findings highlight the importance of an organizational climate for customer orientation and the management of employee – working environment interactions.

## Introduction

Fulfilling the long-term needs and wants of customers has been widely regarded as a key factor in organizational success (Homburg et al., 2009). Customers’ judgment of how organizations meet their needs and wants is largely influenced by the service received from the frontline employees, as they are the first and, quite often, the only representation of a service firm (Chuang et al., 2012; Hartline et al., 2000; Keane et al., 2024). Therefore, how to manage frontline employees and motivate them to deliver superior quality service to customers, hence ultimately enhancing organizational performance, is a crucial issue for companies in the service sector (Li et al., 2024; Sürücü et al., 2022). One of the key concepts that researchers have identified in this domain is customer orientation. Two customer orientation constructs have been widely studied in parallel. On the firm level, Organizational Customer Orientation (OCO), an organization’s continuous and proactive disposition that puts the interests of customers first (Narver and Slater, 1990; Stocchi et al., 2022), has been examined particularly for its impact on firm’s performance (e.g., Frambach et al., 2016). On the individual level, Employee Customer Orientation (ECO), an employee’s work value that presents the amount of his or her affect for or against customers, has been widely studied for its influence on the employee’s performance (Axtell et al., 2007; Brown et al., 2002; Schuh et al., 2011; Mahmoud et al., 2020).

However, the prevalent research design in the literature has focused on either OCO or ECO with regard to their respective impacts on firm or employee performance. Although Cross et al. (2007), Liao and Chuang (2004), and Lam et al. (2010) all called for a more fine-grained perspective of how these two customer orientation constructs may interact with each other, there has been little research that integrates different levels of customer orientation. Moreover, the literature offers limited knowledge on how customer orientation on the organizational level can be diffused to ultimately drive frontline employees’ job performance. Since frontline employee performance is particularly important to the customers as it positively relates to customer satisfaction and loyalty (Wieseke et al., 2012), the organization’s long-term success actually depends on individual performance by employees at the frontline. Therefore, it becomes particularly salient to examine how customer orientation at the organizational level (i.e., OCO) can be disseminated among frontline employees to achieve a high-quality service delivery.

Another reason for the investigation of the impact of OCO on employee performance is that, even among the limited number of existing studies on this relationship, the results are inconsistent and mixed. For example, Liao and Chuang (2004) have found a positive direct relationship between OCO to employees’ job performance among restaurant managers and employees. However, the study by Cross et al. (2007) fails to establish such a relationship with business-to-business salespeople. The current inconsistent results indicate that, for OCO to exert a positive effect on frontline employees’ job performance, some mediating and moderating mechanisms are present. Accordingly, our study aims to investigate the mediating and moderating mechanisms for the relationship between OCO and the employee performance. Our study intends to answer the question of how OCO can be disseminated to influence frontline employees’ job performance and under which condition the OCO can be more or less influential on the performance.

We argue that social learning theory (Bandura, 1977) provides a strong theoretical foundation to uncover the mediating and moderating mechanisms. Social learning theory posits that a reciprocal interaction of cognitive and environmental determinants provides a more adequate explanation of employee behaviors (Bandura, 1977). Social learning theorists argue that the person and the environment do not function as independent entities but interactively impact each other to determine individuals’ cognitions and behaviors (Davis and Luthans, 1980). Two types of individual learning were proposed (Bandura, 1977): reinforcement and vicarious learning. Reinforcement learning refers to people’s learning from the consequences of their behavior; for example, employees learn that the customer-oriented behavior can be rewarded or punished from the company’s job descriptions, rules, and policies. Vicarious learning refers to people’s leaning from observing the behaviors of others; for example, employees learn from their managers for customer-oriented or non-oriented behaviors because doing so may enable them to avoid needless and costly errors (Lam et al., 2010). Therefore, the employees’ cognitions and behaviors in the organization are the learning results from the interactions with their working environment.

Drawing on social learning theory and person-organization fit theory, we proposed that ECO acts as the mediating effect for the relationship of OCO on employee job performance and the employee’s personality trait of openness to experience moderates this relationship. ECO refers to employees’ work value and tendency to meet customer needs in their work. On the basis of social learning theory, employees’ own work values and attitudes (i.e., ECO) not only interact with facets of situations such as incentive systems and norms within the organization (i.e., OCO) to affect their behavioral responses, but also are regulated by the environmental cues. Moreover, person-organization fit theory suggests that employees with characteristics that matched those required by their organization’s strategy would enhance their job performance and lead their organizations to greater levels of OCO (Kristof-Brown et al., 2023; Raddatz, 2024). PO fit thus serves as a construct which drive the compatibility between employee and the organization in fulfilling OCO. That is, employees’ own beliefs of customer orientation depend on whether the organization has the overarching value, policies and practices to reward employees to present customer-oriented behaviors and the influence of observing the managers for their customer orientation.

Although previous research defines ECO as individuals’ predisposition, more and more scholars recognized that ECO can be enhanced or decreased in the workplace environment (Kirca et al., 2005), for example, the studies of Zablah et al. (2012) and Kirca et al. (2005) found that ECO is associated with employees’ perception of the organization’s market orientation and their roles within the organization. Also, noted by Liao and Chuang (2004), ECO might depend on their interpretation about the clues from their surrounding work environments.

Social learning theorists generally recognize that employees’ behavior is a joint result of situation and individual differences (Chatman, 1989). Moreover, extant inconsistent and mixed results for the relationship of OCO and ECO (e.g., Cross et al., 2007; Jones et al., 2003; Liao and Subramony, 2008) have also indicated that some individuals may be more prone to customer orientation than others. Taken together, we argued that individual differences (e.g., employees’ personality trait) may present a boundary condition for the causal chain of OCO – ECO – employees’ performance. Especially, also drawing on social learning theory, we propose that employees’ personality trait of openness to experience acts as a moderator on not only the direct effect of OCO on ECO but also the indirect effect of OCO on employees’ performance through ECO. Individuals’ openness to experience describes the extent to which individuals are curious, imaginative, amenable to new ideas, and willing to learn. Social learning theory and extant literature indicate that individuals’ learning and learning style are related to their personality trait of openness to experience (Dragoni et al., 2009) and we thus propose this moderating effect.

Our research contributes to the literature by developing and testing a moderated mediating model that integrates customer orientation on both organizational and individual levels, employees’ personality trait, and employees’ job performance. Our study can make the following contributions. First, the study investigates the neglected link between OCO and employees’ job performance. It advances the literature in illustrating a mediating mechanism of employees’ own values of customer orientation. Our study presents a fine-grained and holistic perspective that integrates different levels of customer orientation. Second, the study sheds new insights into how service workers’ personality trait (i.e., openness to experience) may present a boundary condition for the direct relationship of OCO on ECO and the indirect relationship of OCO on employee job performance. Third, this study also enhances the theorists’ arguments that employees’ behavior is influenced by both working context and individual differences. That is, employees’ job performance is a behavior of a continuous, reciprocal interaction between environmental (i.e., OCO), cognitive (i.e., ECO), and personal (i.e., openness to experience) determinants.

## Conceptual framework and hypotheses

**Figure 1:**
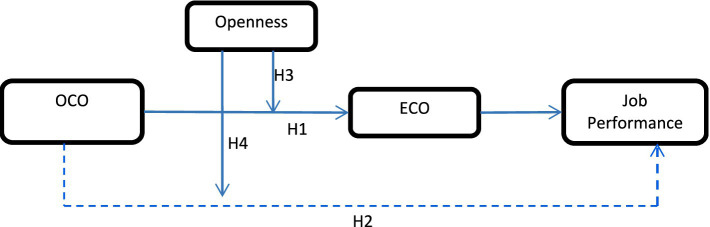
Conceptual model and hypotheses. OCO = Organizational Customer Orientation; ECO = Employee Customer Orientation; Openness = Employee’s Openness to Experience. 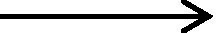: direct effect. 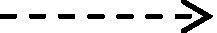: indirect effect.

Overall, we propose a framework that integrates the mediating effect of ECO and the moderating effect of employees’ personality (i.e., openness to experience) on the effect of OCO on employees’ job performance. We expect that OCO positively affects employees’ job performance through the impact on ECO. Moreover, OCO and ECO will have a stronger relationship for employees with higher personality traits of openness to experience. Also, the OCO – ECO – employees’ job performance chain has a stronger relationship for employees with this personality (see Figure 1).

Organizational Customer Orientation and Employee Job Performance.

OCO refers to an organization’s continuous and proactive disposition that puts the interests of customers first (Narver and Slater, 1990). It is a set of beliefs that customer needs and satisfaction are the most important concern for the whole organization, and the organization needs to sufficiently understand its customers and continually find ways to deliver superior customer value (Grizzle et al., 2009). Consistent with the extant literature, i.e., Grizzle et al. (2009), Cross et al. (2007), and Liao and Chuang (2004), we refer OCO to employees’ shared perceptions of the organization’s focus on customer need satisfaction as implemented by managers (Grizzle et al., 2009, p. 1229). This is because, first, although OCO can be seen as an organizational culture and commitment that puts the customer first, whether the frontline employees who have daily contacts with customers have realized this or not is another issue. Even if the senior leadership team or managers recognize customer orientation, this might not have to be related to employees’ recognition of customer orientation across the organization. For example, previous research (i.e., Jones et al., 2003) has found that managers’ perception of OCO is not correlated with employees’ perception of OCO. Second, both the seminal studies of Grizzle et al. (2009) and Liao and Chuang (2004) have indicated that the frontline employees, rather than the managers, are more appropriate to evaluate the degree of the customer orientation on the organizational level. And third, this study explores the effect of OCO on employees’ job performance; therefore, employees’ perception of OCO has more rational and logical meaning for their job performance.

Within the limited number of empirical studies of OCO and employees’ performance, some have found the positive association between them (Liao and Chuang, 2004). According to the social learning theory (Bandura, 1977), employees’ perception and feel about certain aspects of the working environment can directly influence how they behave and work. Liao and Chuang (2004) noted that the development of employees’ attitudes and the understanding of expectations concerning their behaviors depend on their interpretation about the clues from their surrounding work environments. Therefore, when employees have perceived that, within the whole organization, creating superior customer value is expected, desired, and rewarded, they are more likely to reflect this perception during the work so as to provide high quality service to the customers. In contrast, when the situation signals that putting the customer first is discouraged, employees should show less effort to perform their job to a high quality because the environment constrains such a performance. Thus, employees’ perception of OCO is significantly correlated with their job performance.

However, as argued previously, the extant literature on the relationship between OCO and employees’ job performance offers inconsistent results. We argue that one possible explanation for the inconsistent result is that, for OCO to exert a positive effect on employees’ job performance, some mediation and/or moderating mechanism is present. First, we propose that the customer orientation of employees may act as a mediating factor between OCO and employees’ job performance.

### Mediating role of employee customer orientation

ECO refers to the employee’s tendency to meet customer needs in their work, and is defined as the “*employee’s work value that captures the extent to which employees’ job perceptions, attitudes, and behaviors are guided by an enduring belief in the importance of customer satisfaction”* (Zablah et al., 2012, p. 24). It represents an employee’s work value that refers to the amount of his or her affect for or against customers. Therefore, also noted by extant studies, ECO can be enhanced or decreased in the workplace rather than an individual’s enduring trait (Franke and Park, 2006; Zablah et al., 2012). Employees with high customer orientation are more likely to understand the customers sufficiently so as to continually create superior values for them. The extant studies have found a positive direct relationship between ECO and frontline employees’ job performance (Babakus et al., 2009; Brown et al., 2002; Franke and Park, 2006; Menguc and Boichuk, 2012). This is because that, placing the highest priority on meeting customer needs, customer-oriented employees can understand and satisfy customers’ needs more effectively and communicate with their customers more efficiently (Donavan et al., 2004), while helping them diagnose problems and developing functional long-term relationships (Homburg et al., 2009). Indeed, extant literature has noted that employees’ job performance, especially in the service sector where this research was conducted, is mainly reflected through individuals’ desire to help customers assess their needs, maintain a consistent level of kindness toward customers, and deliver solutions to customers’ problems (Babakus et al., 2009). Therefore, employees with high customer orientation should have a high quality performance in their jobs.

We propose that ECO may act as a mediating role between OCO and employees’ job performance. First, the person–situation interaction theory (Schneider, 1987) proposes that employees’ aspects such as values and attitudes interact with facets of situations such as incentive systems and norms to affect their attitudinal and behavioral responses (O'Reilly et al., 1991). For example, a number of studies for service organizations have indicated that, when there is a widely spread climate for service focus and orientation within the organizations, employees’ perception and individual performance of customer service can be motivated (Liao and Chuang, 2004). Therefore, when an organization has the policy and practice to encourage and expect customer orientation attitudes and behaviors, individual employees are more likely to adopt a customer orientation value as a result of situational and environmental influences.

Second, a social learning perspective also suggests that ECO, like other working value items, is regulated by environmental cues (Jones et al., 2003). The study of Yoo and Arnold (2016) indicates that the organizational support is positively associated with frontline employees’ customer-oriented attitude. As explained before, social learning theory proposes employees’ behavior as a continuous, reciprocal interaction between cognitive, behavioral, and environmental determinants. On one side, employees may enhance their own customer orientation through enhancement or consequence learning, that is, their own attitudes and behaviors of customer orientation can be rewarded or punished, depending on their perception of whether the organization has the policies, practices, and procedures that reward, support, and expect concerning customer orientation or not. Whilst on the other side, employees’ own customer orientation may also be enhanced through vicarious learning, that is, observing their managers who present various customer orientation behaviors may also help frontline employees engage in more customer orientation. Moreover, Davis and Luthans (1980) pointed out that the main focus of social learning theory is to investigate the mediating effect that covers cognitive process may have on an observable sequence of events (p. 285). So that, the cognitive processes (i.e., ECO), representing the personal variable, play an important mediating role and are the interaction with environment (i.e., OCO) and behavior (i.e., job performance). Therefore, when customer orientation is becoming more prevalent through both organizational policy and managers’ behaviors across the organization, it may lead to the increase of ECO and ultimately to a better job performance. Given the aforementioned relationships within the OCO – ECO – employees’ job performance chain, we posit that OCO positively relates to employee job performance through ECO.

*Hypothesis 1:* Organizational customer orientation has a positive effect on employee customer orientation.

*Hypothesis 2:* Employee customer orientation mediates the relationship between organizational customer orientation and employees’ job performance.

### Moderating role of employee openness to experience

As discussed in the previous section, the relationship between OCO and ECO can be explained well by social learning theory; however, the extant studies on OCO with ECO have presented mixed and inconsistent results. For example, Liao and Subramony (2008) found that, in their study of a global consumer durables manufacturer, the customer orientation of the organization is positively associated with the customer orientation of salaried employees. However, Cross et al. (2007) with business-to-business salespeople has found that their perception of OCO can not lead to the increase of their own customer orientation. The same results have also emerged in the study with salespeople from grocery retailers by Jones et al. (2003) that investigates the relationship between OCO and ECO. Regardless of how customer orientation attitudes and values are acquired by employees, there is clear evidence that some individuals are more prone to customer orientation than others (Brown et al., 2002; Liao and Chuang, 2004). Taken together, the explanation for the inconsistent findings produced by extant studies is that OCO may not be effective for ECO and ultimately for job performance when employees are individually different, which implies the moderating role of personality traits. In fact, employees with different personalities and characteristics surely present different values and attitudes by encountering environmental factors. Extant studies on customer orientation (e.g., Grizzle et al., 2009; Román and Iacobucci, 2010) have noted that individual difference in customer orientation is an important but under-researched issue.

We posit that the positive effect of OCO on ECO depends on employees’ own characteristics. Especially, we argue that employees’ personality trait of openness to experience will positively moderate the effect of OCO on ECO. That is, although in general OCO positively relates to ECO, especially for those employees who have the personal characteristics of opening to new experience, OCO has no significantly positive relationship with ECO for employees who are at the low end of openness to experience. Our argument is consistent with social learning theory in that the person and the environment determine each other in a reciprocal manner that acts as an interactive effect between them and somehow combine to determine individuals’ cognitions and behaviors.

Openness to experience refers to a tendency to enjoy new experiences and new ideas. Open people are normally fascinated by novelty and innovation as they are imaginative, original, witty, and intelligent, while people at the other end are conventional and find comfort in the familiar or routine work (Barrick and Mount, 1991). Accordingly, employees high in openness to experience prefer novelty, variety and intense experience and employees at the low end prefer the familiar, routine, and traditional experience. Moreover, individuals high in this trait may engage more in interventions with an enhanced perception of their efficacy. Wanberg and Banas (2000) have found that employees high in openness are more likely to exhibit certain tendencies of values such as positively viewing workplace transitions and changes in contemporary work environments.

We argue that employees’ openness to experience can enhance the effect of OCO on ECO for the following reasons. First, as we discussed previously, the relationship between OCO and ECO sits well with social learning theory. The extant literature indicates that individuals’ learning and learning style are related to their personality (Dragoni et al., 2009). Especially, openness to experience describes the extent to which individuals are curious, imaginative, amenable to new ideas, and willing to learn. Moreover, the studies of Grizzle et al. (2009) and Román and Iacobucci (2010) confirm that OCO is a contextual factor that can activate employees’ own characteristics, in that when employees perceive high customer orientation among their working environment, it provides opportunity for individuals’ personality traits to manifest. Therefore, employees’ openness to experience provides an important boundary condition for the effect of OCO on ECO. That is, for employees who are higher in openness, OCO should have a stronger effect on their individuals’ customer orientation because this personality encourages them to accept new environmental encouragement. Under the current context, the environmental encouragement refers to not only the organizational policy and practice for customer orientation (i.e., enhancement learning), but also managers’ customer-oriented manner (i.e., vicarious learning). Therefore, since individuals high in openness are more willing to make adjustments to themselves when they are exposed to new situation or environmental encouragement, their openness personality can enhance the effect of OCO on ECO.

Second, OCO is defined that organizations need to continuously and proactively find ways to provide superior customer values. In order to make ECO positively related to ECO, customer-oriented organizations, especially within the current study context of service sector, require employees to be witty, intelligent, and imaginative, so as to find different ways to meet customers’ needs. Prior theory and research suggest that openness to experience is the trait that may be most relevant for employees’ creative behaviors (Shin et al., 2012). In contrast, employees with low openness to experience trait are routine, conservative, and conventional. They are reluctant or less adaptive to adjust themselves and find new ways to solve customer problems (Homan et al., 2008). That is, the OCO can not positively lead to ECO because of personal constraint. In sum, for employees who are high on openness to experience, they are more likely to manifest a certain predisposition that enhances the effect of OCO on ECO in a work setting, especially the work setting (i.e., encouragement of customer orientation by the organization) to allow for and encourage the demonstration of that predisposition.

*Hypothesis 3:* Organizational customer orientation has a stronger relationship with employee customer orientation for employees with higher openness to experience.

Given that ECO acts as a mediating effect of OCO on employee job performance. Meanwhile, the employee openness to experience acts as a moderating effect of OCO on ECO. Thus, we posit the following moderated mediating effect:

*Hypothesis 4:* Organizational customer orientation has a stronger indirect relationship with employees’ job performance through employee customer orientation for employees with higher openness to experience.

## Method

### Study context

We test our conceptual model in the context of Nigeria. Nigeria is a West African nation and the largest economic power in Africa. Nigeria has experienced rapid structural change over recent decades, moving beyond a natural resources export base toward a diversified economy in which services account for a substantial share of output and employment. While nature resource has long played a prominent role in national revenues, domestic economic activity has increasingly shifted toward finance, telecommunications, real estate, and other consumer-facing services. Concurrent policy initiatives aimed at digitalization, financial inclusion, and urban development have further accelerated the expansion of service firms that interact with customers in Nigeria (Maduka, 2016). Within this broader transformation, financial services, insurance, telecom solutions, and estate services form an ideal empirical setting for our study.

First, most studies investigating customer orientation are in Western developed countries (Brown et al., 2002; Cross et al., 2007; Grizzle et al., 2009; Homburg et al., 2009), or Eastern countries (Zhou and Li, 2010). No research of customer orientation has been studied with service organizations in Africa. Moreover, although many scholars indicate the important effectiveness of organizations in developing and emerging economies (Gunz and Peiperl, 2007), the extant literature is surprisingly lacking for studies of customer orientation in African countries. Second, ethnic diversity is the most typical characteristic among African countries and companies (Kamoche, 1997). With the largest population in Africa, Nigeria also has the richest ethnic diversity (i.e., more than 250 ethnic groups) among all African countries. Thus, Nigeria has been typically and widely accepted as a representative case for African countries (Gomes et al., 2012). Third, having initiated extensive economics reform programmes in recent years, the services industry (i.e., current study context) has been rapidly developing throughout the country; but how well the frontline employees can deliver high quality service is still a challenge.

### Samples and procedure

We collected our data from both service employees and their immediate supervisors working in nine service organizations. These organizations are the service companies that provide financial services, insurance, telecom solution, estate services, etc. A total of 1,077 employees form the total population of the study. In order to reduce concerns of common method bias and to have good reliabilities, two waves of data collection method have been conducted (Hanif et al., 2025; Zhao et al., 2022). In the first wave, a cover letter explaining the purpose of the study was presented to 657 employees (61.0% of total population) to ask for their participation in the study. Five hundred and ninety-nine employees agreed to participate in the research, equal to 55.6% of total research samples. A structured questionnaire including the measures of the antecedent (i.e., OCO), moderating variable (i.e., employees’ openness to experience), and demographic questions was compiled in English, the official language in Nigeria, and distributed to those employees. A total of 524 useful responses were collected, with a response rate of 87.5% for the first wave.

The second wave data collection was conducted about three weeks after the first wave survey. In the second wave, we only reached those employees who participated in the first wave administration and provided useful feedback. However, within the 524 respondents participating in the first wave survey, 36 employees were either not in their offices during the second wave or decided not to attend the second wave survey, therefore questionnaires for the measure of the mediator (i.e., ECO) were distributed to 488 employees in their office hours. A total of 393 matched and useful responses were collected, with a wave two response rate of 80.5% and an overall response rate of 65.6%. Respondents who completed both first and second wave surveys were between 21 and 68 years of age, with an average age of 36. The average employee had been with the company for 46.15 months. Women comprised 47.1% of the respondents.

Based on the completed 393 responses, at the third point of time, their immediate supervisors (35 in total) were contacted for their ratings of the subordinates’ job performance (i.e., dependent variable). The immediate supervisors are normally the department heads responsible for employees’ daily work, so they are more appropriate to evaluate their subordinates’ job performance than other persons within the company. All these supervisors kindly provided feedback, resulting in a response rate of 100%.

### Measures

All questions, unless otherwise stated, were scored on a seven-point scale with response options ranging from 1 (‘strongly disagree’) to 7 (‘strongly agree’).

*Organizational customer orientation (OCO)*. OCO was measured with a 10-item scale original created by Narver and Slater (1990) and developed by Grizzle et al. (2009). As we have argued before, we follow the studies such as Grizzle et al. (2009), Liao and Chuang (2004), and Cross et al. (2007) to measure employee perception about OCO. The measurement has been adopted in different nations with varied cultural background. Employees were asked to consider their company or branch managers in the work and indicate the extent to which they agreed or disagreed with some descriptions about OCO toward customers. Sample items include “Our managers constantly make sure that the employees are trying their best to satisfy customers,” and “Our managers assess customer satisfaction regularly.” We removed one item (item #10) due to low item-to-total correlation. The Cronbach alpha is 0.92. Removing this item did not change significantly any subsequent hypotheses testing results.

*Employee customer orientation (ECO)*. ECO was measured by using a 12-item scale developed by Brown et al. (2002). This measure comprises a two-dimension scale including two components: customer orientation enjoyment and needs. This 12-item measurement has been used predominantly in empirical research in a variety of contexts across different cultural background. Sample items are “I get satisfaction from making my customers happy,” and “I keep the best interests of the customer in mind.” The Cronbach alpha is 0.89.

*Openness to experience*. We measured employees’ openness to experience with a 5-item scale (Brown et al., 2002) on a seven-point scale (1 = “never happen”; 7 = “always happen”). Sample measures include “Frequently feel highly creative,” and “More original than others.” The Cronbach alpha is 0.86.

*Employees’ job performance*. In order to correctly measure certain employees’ job performance, we explicitly instructed their immediate supervisors to rate their job performance. Supervisors were asked to recall the service and overall performance of their subordinates who had participated in this study and evaluate them by using a 4-item measurement adopted from Babakus et al. (2009). This measurement was originally used in the banking industry and has been widely applied in other empirical studies. Items include “He/she is a top performer,” “His/her performance is in the top 10%,” “He/she has been rated consistently as a star performer,” and “He/she consistently delivers better quality service than others.” The Cronbach alpha is 0.94.

Although the use of a shorter version of the job performance scale was intended to enhance supervisor cooperation and encourage more thoughtful responses, given that each supervisor was required to evaluate multiple subordinates, a limitation of the present study is the use of short versions of the scales (e.g., job performance, openness to experience). While these measures have been validated and preserve acceptable construct validity, they may not fully capture the richness of the constructs compared to the longer forms. Future research may consider employing the full versions of scales (e.g., Openness from the Big Five Inventory; John et al., 1991) or multidimensional performance measures (Mohammed et al., 2002) to provide a more comprehensive assessment.

The variables used in this study are listed in Table 1.

**Table 1:** List of the questionnaire items.

Variables	Sample items	Source
Organizational customer orientation (OCO)	“Our managers constantly make sure that the employees are trying their best to satisfy customers” “Our managers assess customer satisfaction regularly”	Grizzle et al. (2009)
Employee customer orientation (ECO)	“I get satisfaction from making my customers happy” “I keep the best interests of the customer in mind.”	Brown et al. (2002)
Openness to experience	“Frequently feel highly creative,” and “More original than others”	Brown et al., 2002
Employees’ job performance	“He/she is a top performer,” “His/her performance is in the top 10%,” “He/she has been rated consistently as a star performer,” and “He/she consistently delivers better quality service than others”	Babakus et al. (2009)

## Analysis and results

### Measurement validities

We applied confirmatory factor analyses (CFA) to test the validities of our measurement scales. We first ran a CFA model with the proposed four factors (employee customer orientation was modeled as a second order latent variable consisting of two dimensions: enjoyment and need). Model modification index suggested a few pairs of error terms of items within the same latent variables (i.e., organizational customer orientation, and the enjoyment dimension of employee customer orientation) to be correlated. This model achieved good model fit: χ^2^/*df* = 2.62; CFI = 0.92; TLI = 0.91; RMSEA = 0.06; SRMR = 0.05. We then compared this model with a number of models with three factors (by combining any two latent variables into one) and with the one factor model. Table 2 presents the model fit indices of all models. It clearly shows that the four-factor model is a better model than all the others.

**Table 2:** Confirmatory factor analysis.

Model	χ*^2^/df*	CFI	TLI	RMSEA	SRMR
Proposed four-factor model	2.62	0.92	0.91	0.06	0.05
Organizational customer orientation and employee customer orientation combined	3.51	0.87	0.86	0.08	0.13
Organizational customer orientation and openness to experience combined	4.41	0.82	0.82	0.09	0.10
Organizational customer orientation and job performance combined	6.33	0.72	0.71	0.12	0.13
Employee customer orientation and job performance combined	3.54	0.87	0.86	0.81	0.15
Employee customer orientation and openness to experience combined	3.36	0.88	0.87	0.08	0.11
Job performance and openness to experience combined	4.62	0.81	0.81	0.10	0.12
One factor	8.69	0.59	0.60	0.14	0.16

Table 3 presents the means, standard deviations, AVE, squared AVE, and correlations of all variables. It shows that all variables’ AVE scores are higher than the threshold of 0.50, and the squared AVE scores are higher than the correlations of any pairs of variables. Therefore, the discriminant validities are achieved for all variables.

**Table 3:** Descriptive statistics and discriminant validities.

	1	2	3	4
1. Organizational customer orientation	0.74			
2. Employee customer orientation	0.36^**^	0.95		
3. Openness to experience	0.37^**^	0.42^**^	0.74	
4. Job performance	0.12^*^	0.35^**^	0.28^**^	0.89
Mean	5.89	6.18	5.87	5.11
SD	0.88	0.65	0.86	1.03
AVE	0.55	0.90	0.55	0.80
Cronbach’s alpha	0.92	0.89	0.86	0.94

Diagonal represents the squared AVE. **p < 0.01. *p < 0.05.

### Hypotheses testing

Moderated mediation path modeling was used to test hypotheses with M-PLUS 6.0 due to its strength in testing complex models involving mediations and moderations as being compared to traditional regression analyses. The M-PLUS program can also deal with non-independence of ratings by leaders for job performance through the command of “type = complex.” Doing so takes into account non-independence of the endogenous variable of supervisor ratings of job performance. We standardized all variables for the path modeling. The model based on our conceptual framework achieved good fit: (χ^2^/*df* = 2.37; CFI = 0.98; TLI = 0.97; RMSEA = 0.06; SRMR = 0.02). Table 4 shows the coefficients of the direct and indirect paths of the model. We ran another model in which the direct paths from OCO to the outcome variables were added. This more complex model did not produce better model fit and did not change the coefficients and their significance levels of the existing paths. And none of these added direct paths was statistically significant. Therefore, we report the results of the more parsimonious model.

**Table 4:** Path modelling estimation of coefficients.

	Path coefficients	Indirect paths coefficients (via employee CO)
OCO – ECO	0.28**	
Openness to experience – ECO	0.33**	
Openness to experience x OCO – ECO	0.16**	
Openness to experience – Job performance	0.16*	
ECO – Job performance	0.28**	
OCO – Job performance		0.08**
Openness to experience x OCO – Job performance		0.05*
Openness to experience – Job performance		0.09**

OCO = Organizational Customer Orientation, ECO = Employee Customer Orientation. Standardized coefficients are reported. *p ≤ 0.05. **p < 0.01.

**Figure 2:**
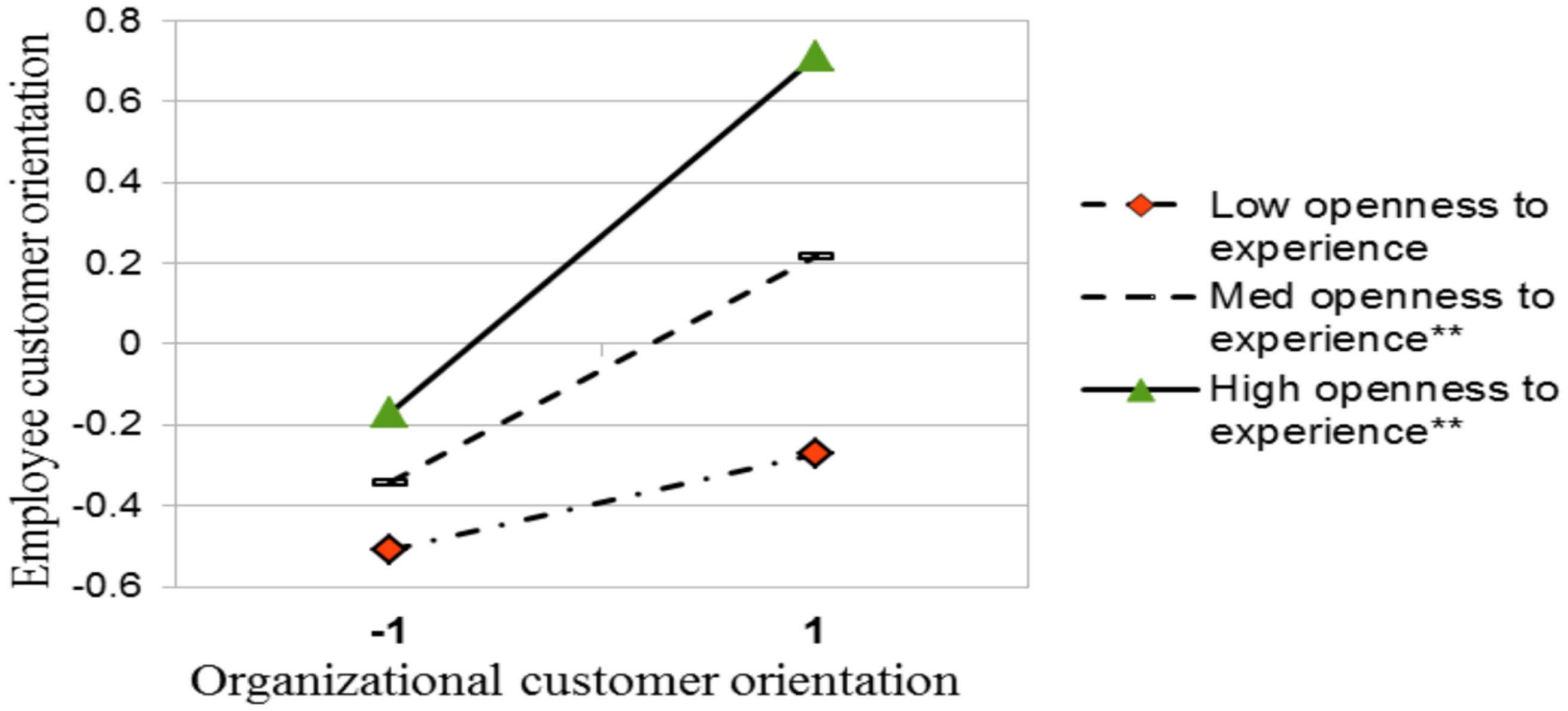
The moderating effect of openness to experience on the relationship between organizational customer orientation and employee customer orientation. **p < 0.01.

Hypothesis 1 predicts that OCO has a positive effect on ECO. H1 is supported (*β* = 0.28, *p* < 0.01). Hypothesis 2 states that ECO mediates the relationship between OCO and employees’ job performance. H2 is supported (β = 0.08, *p* < 0.01). We predicted (in H3) that openness to experience positively moderated the relationship between OCO and ECO. Table 4 shows that the interaction between OCO and openness to experience positively predicts ECO (β = 0.16, *p* < 0.01). We plotted Figure 2 to illustrate this interaction effect. It shows that when openness to experience is higher (one standard deviation above mean), the positive relationship between OCO and ECO is stronger (β = 0.44, *p* < 0.01) than when openness to experience is lower (one standard deviation below mean; β = 0.12, *ns*). On average (when openness to experience is mean), the relationship is significantly positive (β = 0.28, *p* < 0.01).

**Figure 3:**
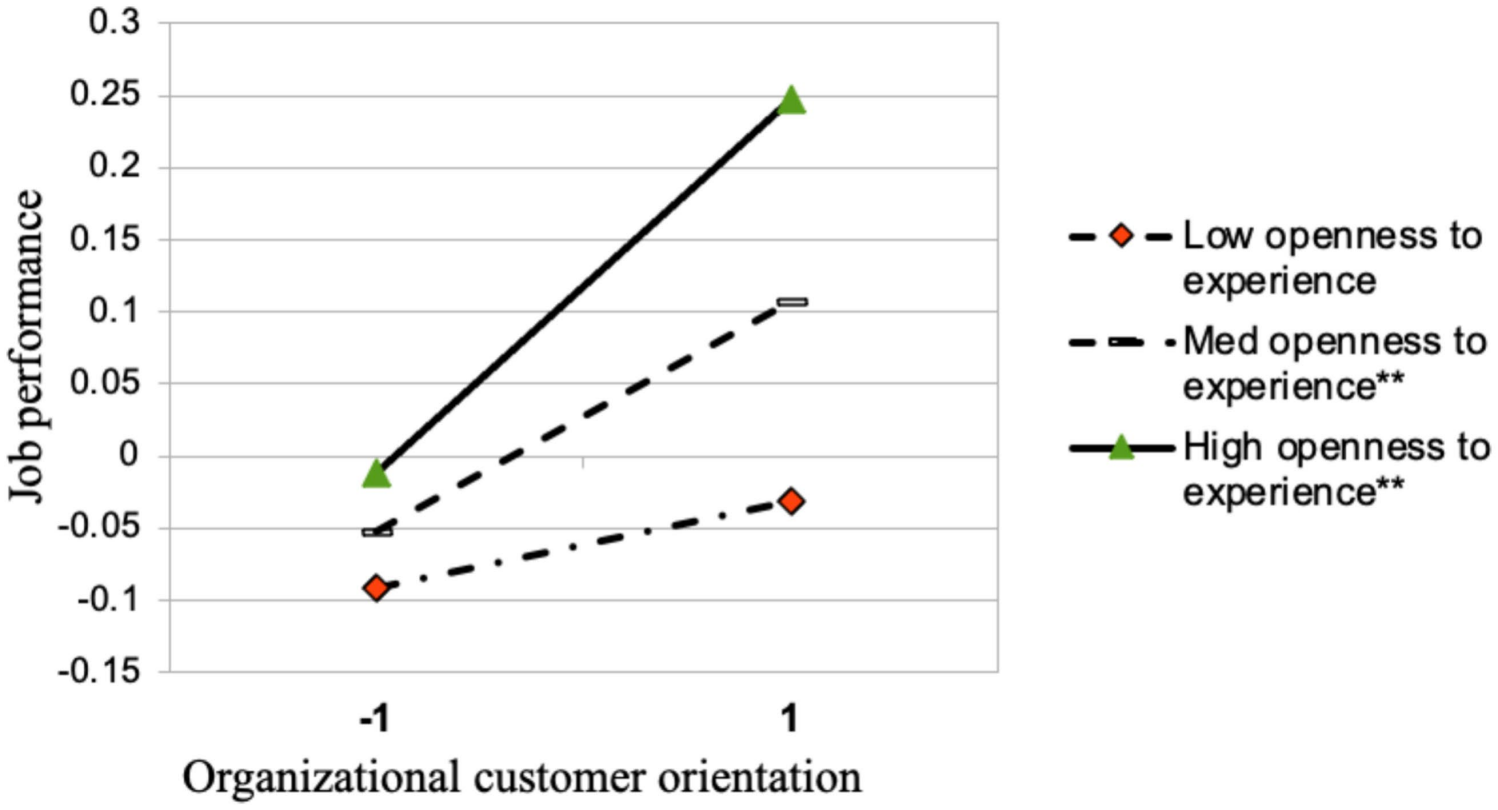
The moderating effect of openness to experience on the indirect relationship between organizational customer orientation and job performance via employee customer orientation. **p < 0.01.

Hypothesis 4 predicts that OCO has a stronger indirect relationship with employee job performance through ECO for employees with stronger openness to experience. To test this indirect effect, we employed 5,000 bootstrap samples. Table 4 shows that the interaction between OCO and openness to experience has a significant and positive indirect effect on job performance (β = 0.05, *p* < 0.05,). We plotted Figure 3 to illustrate this indirect moderation effect. Figure 3 shows that when openness to experience is higher, OCO has a stronger positive indirect effect on job performance via ECO (β = 0.13, *p* < 0.01, LLCI = 0.05, ULCI = 0.22); whilst when openness to experience is lower, such indirect effect is not significant (β = 0.03, *ns*). Thus Hypothesis 4 is supported.

Finally, we also found that openness to experience positively relates to both ECO (β = 0.33, *p* < 0.01) and job performance (β = 0.16, *p* < 0.05).

## Discussion

### Theoretical implications

Extant literature has examined the link between OCO and employees’ performance. The results, however, are mixed and inconclusive (Liao and Chuang, 2004). We argue that such mixed results can be attributed to the misalignment of the perceptions towards customer orientation between management and frontline employees (Jones et al., 2003). In specific, the customer orientation recognized by the management and leadership is not always in line with the employees’ recognition of customer orientation across the organization. In addition, it is more appropriate for the frontline employees, rather than the managers, to evaluate the degree of the customer orientation at the organizational level. We argued that social learning theory and person–organization fit theory provides a strong theoretical foundation to understand how OCO may influence individual frontline employee’s service performance. Our study has identified the mediating role of ECO and moderating role of employee openness to experience in the relationship between OCO and employee job performance.

Addressing this gap, the study makes a number of important theoretical contributions to the literature. First, although customer orientation is argued as a key issue for the success of organizations (Zablah et al., 2012), the study to investigate the relationship between OCO and employees’ job performance is limited. Even within a limited number of those studies, the results are mixed and inconsistent (Cross et al., 2007; Liao and Chuang, 2004). Our study advances the existing literature by identifying a mediation mechanism of employee job-related personal value (i.e., ECO), addressing the question of “how” OCO affects employee outcomes. We contribute to social learning theory by arguing that the way OCO affects employees’ job performance is through enhancing their own customer orientation. Our results are consistent with the findings by Cross et al. (2007) in that, after ECO is controlled for, OCO may not directly predict employees’ job performance. However, our study indicates that OCO can indirectly affect it through employees’ own customer orientation. Therefore, a proactively organizational disposition to put the interests of customers first is still salient as it can provide the environmental encouragement to enhance the customer orientation of employees and ultimately influence their job performance. Moreover, our study also illustrates that employee’s own values and attitudes can be cultivated and should be in organizations as they are positively influenced by OCO.

Second, our study also provides the answer to the extant inconsistent result of the relationship between OCO and ECO. Although sitting well within social learning theory, OCO and ECO has not been found consistently associated within extant studies (see Cross et al., 2007; Jones et al., 2003; Liao and Subramony, 2008). Our study provides the answer to the question under which type of condition the direct relationship of OCO to ECO and the indirect mediating relationship of OCO to employee performance will differ. This research supports the roles played by individual differences (i.e., openness to experience) in the effects of OCO. This study represents one of the first attempts to integrate personality trait with the research of OCO and ECO in efforts to detect boundary conditions. Further, our study also shed additional light on the moderating and mediating mechanisms of the relationship between OCO and employees’ job performance.

Third, openness to experience is argued as a positive antecedent to employee customer orientation and job performance, but some studies (e.g., Brown et al., 2002) failed to support this relationship. Openness to experience is less studied and has less attention than other ‘*positive*’ personality traits (Barrick and Mount, 1991; Fullarton et al., 2014). Some research even mentioned this trait is less welcome and might be a source of annoyance to colleagues and supervisors (Klein et al., 2004). However, the current study indicates that openness to experience is a grateful trait for employees within service organizations. Besides its indirect role to the OCO-ECO-performance chain, our research found that employees’ openness to experience relates positively to both ECO and job performance. This is because highly open employees display intelligence, creativity, and flexible thinking; these traits can positively influence customer service employees to seek methods to meet the customers’ need and solve their problem.

Fourth, the study reinforces the proposal of both situation and individual difference impact on attitude and behavior. Social theorists generally recognize that employees’ behavior is a joint result of situation and individual differences (Chatman, 1989). The empirically tested framework has noted that ECO is the result of both situational cues (i.e., OCO) and individual difference (i.e., employee’s personality trait of openness to experience). Moreover, our study has also indicated that employees’ job performance is not simply a product of individuals’ values and attitudes toward customers, but the results from an interaction between individuals and their situation.

Finally, our findings suggest that the positive effects of customer orientation mainly derived from Western cultures are also generalizable to the Nigerian context. As we argued previously, most studies investigating customer orientation are in Western and Eastern countries. Very few research of customer orientation has adopted social learning theory and studied service organizations in Africa. Situated in Nigeria’s rapidly expanding service economy, frontline work is shaped by two interlocking features that matter for how customer orientation is enacted. First, the structure of service employment is highly heterogeneous (Maduka, 2016). Employees often operate with lean HR systems and acquire skills through day-to-day observation and peer coaching. Second, customer encounters are both multilingual and hierarchical (Endong and Essoh, 2019). Employees routinely switch across local languages and English while working in organizations where supervisory authority is salient, which amplify the influence of manager modeling and team norms. The consistent findings from Nigerian samples extend social learning theory in a new context and demonstrate the importance of customer orientation on both organizational and individual levels.

### Managerial implications

Our research has illustrated some substantial practical implications for service organizations in Nigeria and other emerging economies. First, this research suggests that service organizations do need to have an organizational climate for customer orientation. The employees’ perception of how to serve customers is influenced by not only their job description and organizational policy, but also the messages that senior managers send out. Situated in Nigeria’s rapidly expanding service economy, the structure of service employment is highly heterogeneous (Maduka, 2016). Employees often operate with lean HR systems and acquire skills through day-to-day observation and peer coaching. Therefore, it is salient for the management team to hold an imperative commitment on customer orientation as it may directly impact on employees’ attitude for their own customer-oriented behavior. Service organizations can use this mechanism to educate their service employees how their customer-oriented behaviors would be expected and rewarded by the organization’s policy and practice. For example, managers can guide employees on how the organization values excellent service or deploy certain programs to reward service workers with incentives for their customer-oriented performance. Moreover, although OCO has no direct impact on job performance, it can indirectly influence it through ECO. Therefore, the interaction between OCO and ECO provides recommendation for service organizations on how to improve employees’ service quality.

Second, as we explained before, employees’ social learning is coming from both enhancement learning (e.g., organization’s policy) and vicarious learning (e.g., observation of managers). Even if the organization has job descriptions, rules, and policies to reward customer-oriented behaviors or punish less customer-oriented behaviors, they need to provide training for their managers to make sure that they behave in a customer-oriented manner. Employees encounters are both multilingual and hierarchical in Nigeria (Endong and Essoh, 2019). Employees routinely switch across local languages and English while working in organizations where supervisory authority is salient, which amplify the influence of manager modeling and team norms. Employees learn how to behave from observing those around them and the example behavior that managers provide for their subordinates may be more important than organizational instructions (Davis and Luthans, 1980). Consistent with extant literature, our study also supports that individuals’ high quality performance is influenced by senior managers’ cognition and behaviors (e.g., an organizational cultural and commitment to put customers first), therefore the senior management team and managers need to pay special attention when disseminating customer values and orientation to the frontline employees.

Third, even though service workers may have similar experience of organizational policy and practice of customer orientation, not all employees will react equivalently. Our study provides implications for managers that employees who possess higher level of openness can be expected to respond more favorably to the organization’s continuous and proactive disposition of customer orientation than their counterparts. Moreover, our study also found that employees’ openness may positively directly impact on their job performance. Taken together, the findings provide some useful insights into organizations’ recruitment strategy. For example, in terms of having service workers with favorable response to OCO and high level performance, the recruitment and selection of service workers should incorporate an assessment of the levels of personality traits. Employees with higher openness experience, more curious, flexible, and tolerant of novelty—should be better positioned at advantaged place in the recruitment. Managers also should pay attention to prospective employees’ personality trait of openness to experience for recruitment and job/task allocation. In fact, our findings echo some extant studies in the service industry. For example, the study of Thoresen et al. (2004) indicate that managers may want to consider personality traits that relate to openness and agreeableness when making hiring decisions for sales representatives. Recruitment selection process should also include behavioral interviews and roleplay to test applicants’ curiosity, learning agility, and brief openness.

Fourth, this study has also noted that identifying, monitoring, and developing customer orientation among service workers through training programs can be an effective tactic for maintaining and enhancing employees’ job performance. This is because employees’ own customer orientation acts as not only the antecedent to their ultimate job performance, but also the consequence of organizational stimuli for customer orientation (Schuh et al., 2011). Thus, on one side, managers during the recruitment process may identify prospective employees’ level of customer orientation by designing a difficult customer service scenario that individuals may experience, and asking them to provide solutions; whilst on the other side, managers can foster and cultivate employees’ customer orientation by offering reinforcing the importance of customers to the employees, such as designing a customer-based mission statement or having certain incentives to reward employees’ customer-oriented behaviors.

### Limitations and future research

This study has several limitations that suggest important future research avenues. First, we used Nigeria as the sole context. Although we have explained the contribution of using African samples as most studies for customer orientations are conducted in European, American, and Asian countries, future research might apply and develop our models in other contexts.

Second, we were limited in employing only one employee job-related value, i.e., ECO, as the mediating mechanism. Extant literature (e.g., Hartline et al., 2000; Lam et al., 2010; Román and Iacobucci, 2010) has called for more research on how top management can disseminate customer orientation to each member, particularly the frontline employees who have direct contact with customers. In addition to the increase of ECO, OCO might be cascaded down to frontline employees through other mechanisms. For example, we found that employees learn from the managers to form their customer orientation. Co-workers or peer groups may also be an influencer that acts as the mechanism to impact on ECO and job performance. Therefore, future research can explore other socio-psychological and intro-organizational factors, such as psychological empowerment, organizational identification, peer influence.

Third, our study examined only one boundary condition through which OCO impacts on ECO and employee performance. Future research can explore other boundary effects that *when* OCO impacts. For example, employee differences in terms of their identification with the working organization may also act as the boundary condition under which OCO becomes more or less important. Moreover, although our study provides empirical evidence for the positive effect of OCO on ECO, indirectly and ultimately influencing employee performance, previous studies failed to present consistent results. Therefore, more moderating effects are worth investigating due to the inconsistent results within extant empirical research. Fourth, we borrowed the short version (i.e., four items) to measure employee job performance. Although the results indicated that employees’ performance has a good reliability and validity, future research should adopt stronger longitudinal design and examine multiple forms of employee performance, i.e., service performance, creativity, organizational citizenship behavior to further enhance rigorousness of the study.

Moreover, our research focused on openness to experience as the sole moderator, as it well aligns with the social learning theory. Nevertheless, future research can extend it by examining other personality traits as potential moderators. For example, agreeableness may strengthen the effect of organizational cues on employees’ prosocial responses toward customers, while conscientiousness may amplify the influence of organizational policies and performance standards on customer-oriented behaviors.

Finally, our research focused on openness to experience as the sole moderator, as it well aligns with the social learning theory. Nevertheless, future research can extend it by examining other personality traits as potential moderators. For example, agreeableness may strengthen the effect of organizational cues on employees’ prosocial responses toward customers, while conscientiousness may amplify the influence of organizational policies and performance standards on customer-oriented behaviors.

## Data Availability

The raw data supporting the conclusions of this article will be made available by the authors upon reasonable request.
